# MBD3 Regulates Male Germ Cell Division and Sperm Fertility in *Arabidopsis thaliana*

**DOI:** 10.3390/plants12142654

**Published:** 2023-07-15

**Authors:** Jia Shu, Xiaochang Yin, Yannan Liu, Yingjie Mi, Bin Zhang, Aoyuan Zhang, Hongbo Guo, Juane Dong

**Affiliations:** 1 College of Life Sciences, Northwest A&F University, Yangling 712100, China; 2 School of Advanced Agricultural Sciences, Peking University, Beijing 100871, China; 3 Academy for Advanced Interdisciplinary Studies, Peking University, Beijing 100871, China; 4 College of Chemistry & Pharmacy, Northwest A&F University, Yangling 712100, China

**Keywords:** MBD protein, DNA methylation, pollen, embryo development, male germ cell division

## Abstract

DNA methylation plays important roles through the methyl-CpG-binding domain (MBD) to realize epigenetic modifications. Thirteen AtMBD proteins have been identified from the *Arabidopsis thaliana* genome, but the functions of some members are unclear. AtMBD3 was found to be highly expressed in pollen and seeds and it preferably binds methylated CG, CHG, and unmethylated DNA sequences. Then, two mutant alleles at the AtMBD3 locus were obtained in order to further explore its function using CRISPR/Cas9. When compared with 92.17% mature pollen production in the wild type, significantly lower percentages of 84.31% and 78.91% were observed in the *mbd3-1* and *mbd3-2* mutants, respectively. About 16–21% of pollen from the *mbd3* mutants suffered a collapse in reproductive transmission, whereas the other pollen was found to be normal. After pollination, about 16% and 24% of *mbd3-1* and *mbd3-2* mutant seeds underwent early or late abortion, respectively. Among all the late abortion seeds in *mbd3-2* plants, 25% of the abnormal seeds were at the globular stage, 31.25% were at the transition stage, and 43.75% were at the heart stage. A transcriptome analysis of the seeds found 950 upregulated genes and 1128 downregulated genes between wild type and *mbd3-2* mutants. Some transcriptional factors involved in embryo development were selected to be expressed, and we found significant differences between wild type and *mbd3* mutants, such as *WOXs*, *CUC1, AIB4,* and *RGL3*. Furthermore, we found a gene that is specifically expressed in pollen, named *PBL6*. PBL6 was found to directly interact with AtMBD3. Our results provide insights into the function of AtMBD3 in plants, especially in sperm fertility.

## 1. Introduction

DNA methylation is an older epigenetic modification in eukaryotes that plays many important roles in the regulation of gene expression and embryo development [1]. It is essential for mammalian embryo development, as well as in plants [2]. DNA methylation can occur in all sequence contexts in plants, including CG, CHG (which are symmetrical sites), and CHH (H = A, T, or C), which is considered asymmetrical. Two *met1b* null mutants of OsMET1b in rice showed abnormal seed phenotypes [3]. The *Oscmt3a* mutants exhibited a severe decrease in CHG methylation and pleiotropic developmental abnormalities [4]. Double mutants of maize (*Zmet2*/*Zmet5* and *Chr101*/*Chr106*) result in seed problems [5].

Plant DNA methylation mainly occurs at transposons and repetitive DNA elements [6,7]. Its process involves writer, reader, and editor proteins. The reader proteins can specifically bind methylated CpG dinucleotides through methyl-CpG-binding domains (MBDs) or SET- and ring-finger-associated domains [8,9,10]. MBDs are the earliest proteins found to specifically read and bind methylated sequences, playing important roles in the regulation of DNA methylation, chromatin remodeling, and histone modification [11,12].

The MBD protein family includes 13 members in *Arabidopsis thaliana*, 17 in rice, 14 in *Populus*, 14 in maize, and 6 in wheat [13]. In *A*. *thaliana,* only AtMBD5, AtMBD6, and AtMBD7 have been found to specifically bind to methylated CG sites, whereas AtMBD4 binds to unmethylated DNA [14,15]. *AtMBD4* negatively regulates the phosphate starvation response, altering root morphology [16]. *AtMBD5* performs an important function in maintaining chromatin structure and mitosis [17]. The *AtMBD7* gene is associated with active DNA demethylation and transcriptional gene silencing [18,19]. Other *AtMBDs,* such as *AtMBD8*, result in a delay in flowering time during both long and short days [20]. The mutation of *AtMBD9* results in a significantly earlier flowering than that of wild-type plants [21]. *AtMBD11* displays a variety of phenotypic effects, including abnormal flower position, fertility problems, and late flowering [22]. However, to this day, the functions of the other *AtMBD* members are poorly understood.

In mammals, the *MBD3* knockout is embryonically lethal in mice [23], suggesting the decisive role of *mbd3* in early embryonic development and late embryonic stages. To further explore the function of *AtMBD3* in plants, especially throughout embryo development, CRISPR/Cas9 technology was used to form the *mbd3* mutants of *A. thaliana* and to obtain developmental phenotypes. These mutants will help us understand the regulation of gene expression, particularly in relation to essential and cell cycle genes from the gametophytic to sporophytic phases. Our results provide insights into the function of MBD3 in pollen and embryo development.

## 2. Results

### 2.1. AtMBD3 Was Highly Expressed in Pollen and Seeds

A high degree of amino acid sequence conservation was found among MBD1-13 of *A. thaliana*. The distinct expression profiles of ten MBD proteins were presented from the *A. thaliana* eFP Browser, and the results showed that these proteins had different expression patterns but similar evolutionary characteristics (Figure S1).

**Figure 1:**
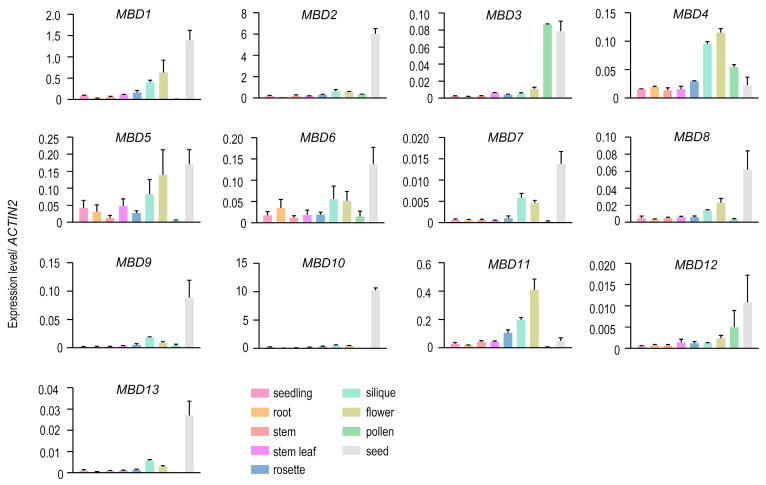
Gene expression of *MBDs* in *Arabidopsis thaliana.* The expression of 13 *MBDs* in 9 tissues of wild-type Col-0 were determined using qPCR. *ACTIN2* was used as an internal control. The error bars represent standard deviations.

A quantitative RT-PCR analysis was performed to evaluate the gene expression of 13 *AtMBDs* (Table S1). Except for *AtMBD4* and *AtMBD11*, most of the *AtMBDs* were highly expressed in seeds. *AtMBD3* was also found to be highly expressed in pollen among all the 13 members (Figure 1).

### 2.2. AtMBD3 Preferred to Bind Symmetric Methylated and Unmethylated Sequences

**Figure 2:**
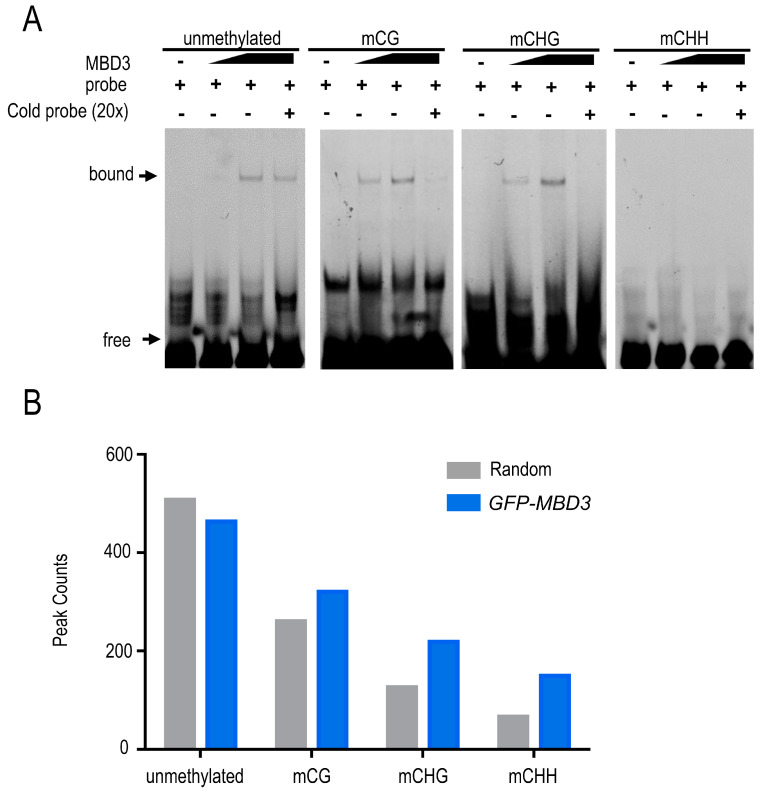
The binding capacity of MBD3 with different mCG, mCHG, mCHH, and unmethylated sequences. (**A**) EMSA results showed that MBD3 protein had the priority to bind mCG, mCHG, and unmethylated sequences. 3′-Fam-labeled substrates (160 nM) were incubated with concentrations of MBD3. The protein concentrations in lanes 1, 2, 3, and 4 were 0, 0.8, 1.6, and 1.6 μM, respectively. For competition analysis, 32 μM of substrates with or without methylation labeling were used as cold probes and incubated with GST-MBD3; and of 3′-Fam-labeled substrates in every fourth lane. For EMSA, at least three independent biological replicates were performed. “Bound” represents the binding band, and “free” represents the free probe. (**B**) ChIP-seq assay was carried out with transgenic *pJIM(Bar)-GFP-linker::MBD3*/Col-0 seedlings, showing the binding potential of AtMBD3 with different methylated regions. The gray column “random” was the control, representing the binding regions in Col-0. The blue columns “GFP-MBD3”represent the binding regions in MBD3.

EMSA (electrophoretic mobility shift assays) was used to detect the binding potential of fusion protein GST-MBD3 and DNA sequences in vitro. The result showed that AtMBD3 had a strong binding preference to symmetrically methylated CG and CHG DNA sequences, and to unmethylated DNA sequences, except for methylated CHH (Figure 2). Although AtMBD3 could bind unmethylated DNA sequences, its binding ability was weaker than that of methylated CG and CHG. The EMSA result of fusion protein MBP-SUVH1 was used to test the labeled DNA sequence (Figure S2, Table S2).

To further determine the binding potential of MBD3 *in vivo*, ChIP assay was performed by expressing *pJIM(Bar)-GFP:MBD3*/*Col-0* in seedlings. Then, a total of 711 peaks were obtained (Table S3), among which 687 had a higher DNA methylation in transgenic plants than in Col-0. A total of 320 peaks had an overlap with mCG methylation regions, 218 peaks overlapped with mCHG regions, and 149 peaks overlapped with mCHH regions. This difference also confirmed the binding preference of the MBD3 protein. 

### 2.3. MBD3 Is Important for Plant Embryo Development

**Figure 3:**
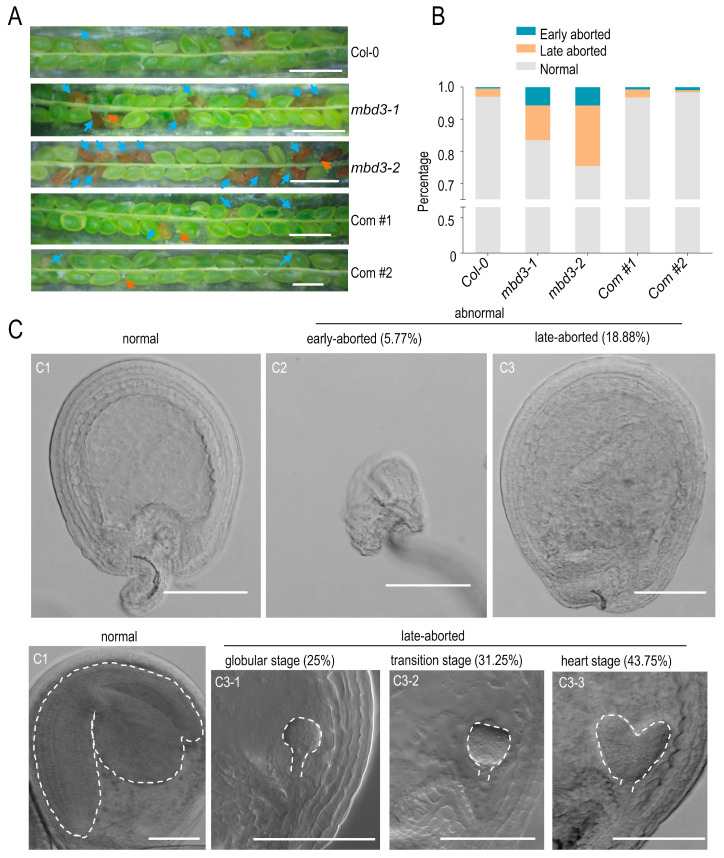
Phenotype of *mbd3* mutant 9 days after pollination. (**A**) Seeds set of Col-0, *mbd3-1*, *mbd3-2*, and complementation lines Com #1 and Com #2. Com #1 and Com #2 represent two individual *proMBD3::MBD3*/*mbd3-2* transgenic plants. Ovules with development problems are indicated by arrows. “Blue “ and “yellow “ arrows represent early- and late-aborted seeds, respectively. Bars = 1 mm. (**B**) Seed abortion rates of Col-0 (*n* = 701), *mbd3-1* (*n* = 1068), *mbd3-2* (*n* = 1144), Com #1 (*n* = 1213)*,* and Com #2 (*n* = 1156) plants. (**C**) Differential interference contrast (DIC) microscopy of whole-mount-cleared ovules showed abnormal cell division. (**C1**–**C3**) Embryo patterns and percentages of normal and aborted embryos in *mbd3-2* siliques (*n* = 96) are shown. (**C3-1**–**C3-3**) Three stages of late-aborted embryos in *mbd3-2*. Bars = 100 µm.

We designed two sgRNAs that target the MBD domain of *MBD3* by using CRISPR/Cas9, and obtained two homozygous transgenic plants, named *mbd3-1* and *mbd3-2* (Figure S3A). Both mutants were found to affect embryo development. In *mbd3-1* (*n* = 1068) and *mbd3-2* (*n* = 1144) siliques, 5.71% and 5.77% of seeds underwent early abortion in plants, respectively. A total of 10.86% and 18.88% of seeds in the *mbd3-1* and *mbd3-2* plants were wrinkled, respectively (Figure 3).

When the normal embryos in *mbd3-2* (*n* = 96) were at the cotyledon stage, 25% of abnormal seeds were at the globular stages, 31.25% at the transition stage, and 43.75% at the heart stage. This means that the embryogenesis defects in *mbd3* plants lead to a delayed embryo development, and that these abnormal seed developments could be divided into three stages. 

### 2.4. MBD3 Mutation Impairs Pollen Development 

To understand why these seeds underwent abortion, we first examined the anther development in Col-0 and *mbd3* mutant plants by using cryo-SEM. The anther and pollen morphologies were not aberrant obviously (Figure S3B). The pollen viability was evaluated by Alexander’s staining, and it was found to produce no effects (Figure S3C). The in vitro germination of pollen was also examined. The germination time, pollen length or quantity of *mbd3* mutant were all found to have no significant difference from Col-0 (Figure S3D).

**Figure 4:**
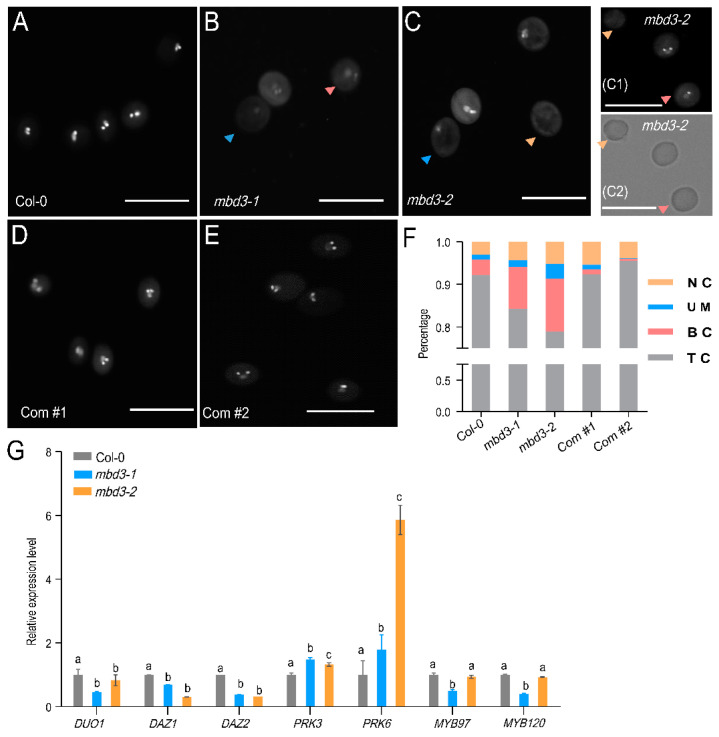
Mutation of *MBD3* impairs pollen development. (**A**–**E**) DAPI staining of mature pollen from Col-0, *mbd3-1, mbd3-2,* Com #1, and Com #2 plants. Different arrows represent abnormal pollen. Red arrows represent binuclear pollen; blue arrows represent mononuclear pollen; yellow arrows represent nonnuclear pollen. Bright area of (**C1**) was zoomed in (**C2**). Bars = 50 µm. (**F**) Phenotypical percentages in uninucleate microspores (UM), binucleate (BC), trinucleate (TC), and nonnucleate (NC) pollen of Col-0, *mbd3-1, mbd3-2,* Com #1, and Com #2 plants (*n* > 300, for each genotype). (**G**) Relative gene expression levels with significant differences in the mature pollen of Col-0 and *mbd3* plants. *ACTIN2* was used as an internal control. Two independent experiments were conducted with similar results. Data from one experiment with four technical replicates. Error bars indicate the mean ± SD; a, b, and c are significant differences (*p* < 0.05, one-way ANOVA).

To further evaluate the effect of *mbd3* mutation on pollen development. Mature pollens from Col-0 and *mbd3* plants were stained with DAPI, and observed with fluorescence microscopy. About 16~21% of pollen from *mbd3-1* and *mbd3-2* plants all suffered a collapse in reproductive transmission. Compared with 92.17% of mature pollen production in Col-0, significantly lower percentages of 84.31% and 78.91% were found in *mbd3-1* and *mbd3-2* plants, respectively. Of them, about 1.61% and 3.45% contained one vegetative nucleus, lacking condensed sperm cells. Approximately 9.82% and 12.42% contained one vegetative nucleus and one condensed sperm cells. Approximately 4.25% and 5.17% did not contain any nuclei. The percentage of two complementary lines, Com #1 and Com #2, approached that of Col-0 (Figure 4). 

A quantitative RT-PCR was performed to dissect the gene expression involved in the male gametophyte development of *mbd3*. The expression of seven genes, including male germline-specific R2R3 MYB transcription factor *DUO1*; two *DUO1* target genes *DUO1-ACTIVATED ZINC FINGER1*/*2 (DAZ1*/*2)*; *POLLEN RECEPTOR-LIKE KINASE3 (PRK3); PRK6*; and MYB transcription factors MYB97 and MYB120 showed a distinct pattern in *mbd3-1* and *mbd3-2* (Figure 4G). Meanwhile, *duo1* mutant and *daz1*/*daz2* double-mutant were found to block generative cell division, producing high percentages of binuclear pollen. 

### 2.5. mbd3 Embryo Development Delay Caused by Asymmetric Divisions 

**Figure 5:**
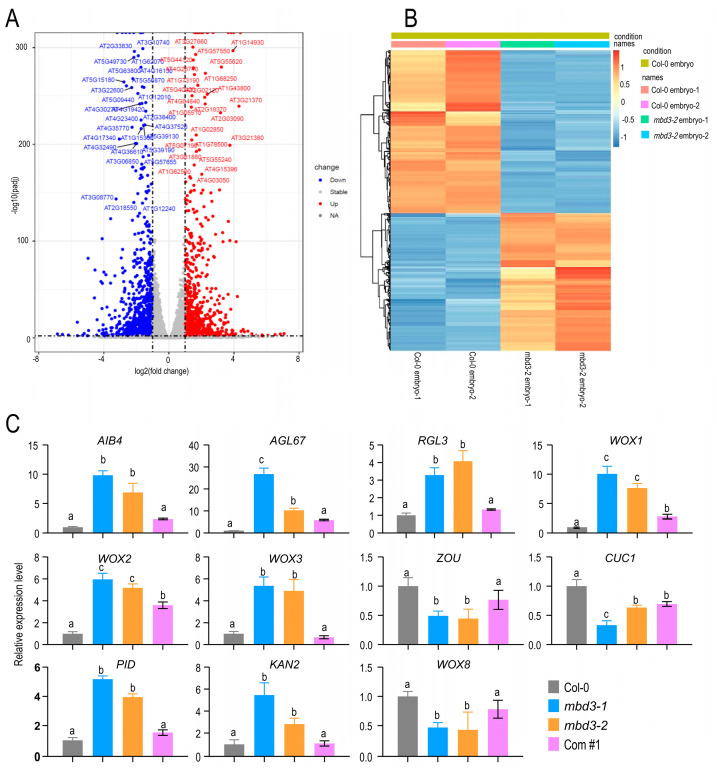
*MBD3* is important for embryogenesis in *A. thaliana*. (**A**) The upregulated and downregulated genes in seeds of Col-0 and *mbd3-2* mutant plants. (**B**) Heat map showing the different expression patterns between Col-0 and *mbd3-2* mutant plants. (**C**) The relative expression level of different transcription factors regulated embryo development by using RT-qPCR analysis. *Tublin8* was used as internal control, given that the expression of *Actins* changed in *mbd3-2*. Two independent experiments were conducted and obtained similar results. Data from one experiment with four technical replicates. Error bars indicate the mean ± SD; a, b, and c are significant differences (*p* < 0.05, one-way ANOVA).

The RNA-seq results analysis of embryos between Col-0 and *mbd3-2* mutant was performed, in which 950 upregulated genes and 1128 downregulated genes were found (Figure 5; Table S4). Some transcriptional factors, which were selected, were reported to be involved in embryonic development (Table S5), such as *CUC1 (CUP-SHAPED COTYLEDON1)* [24], *WOX2 (WUSCHEL-related homeobox2), WOX8*/*STIMPY-LIKE*/*STPL* [25], *PID1 (PINOID)* [26], *ABI4* [27], *RGE1*/*ZOU (RETARDED GROWTH OF EMBRYO1*/*ZHOUPI)* [28], and *AGL67* [29]. The expression pattern of these involved genes indicated that *MBD3* may play an important role in embryo development.

### 2.6. Interaction Protein Screening of AtMBD3

**Figure 6:**
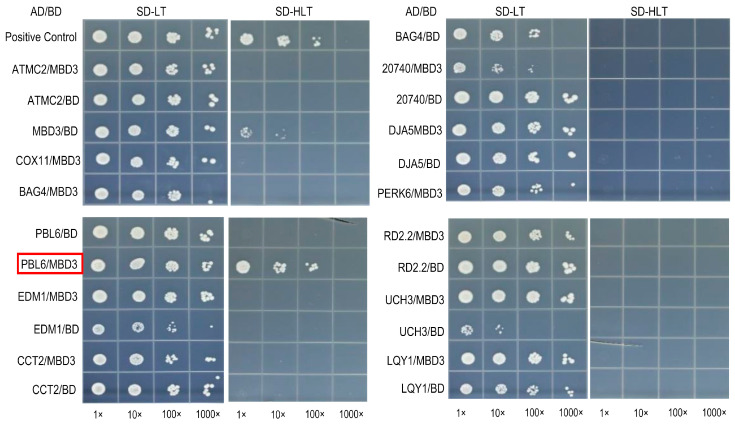
Positive proteins selected to screen interaction with MBD3 by using Y2H. Detailed information on proteins listed in Table 1. Proteins that interacted with AD or BD were negative controls to detect self-activated. The red rectangle is the positive result. The empty wells on the images signify no direct interaction.

Y2H and IP-MS were used to screen the interaction proteins of MBD3. BD-MBD3 plasmid was used as bait, while the prey library was the *A. thalinana* cDNA library. The resulting progenies were selected on SD/-Leu/-Trp/-His/-Ade plates by adding β-galactosidase to eliminate false positives. A total of 69 positive progenies were obtained (Table S6). PCR was adopted to verify these proteins. Through sequencing and blasting the 69 verified proteins, 12 repeatedly identified positive proteins were selected and listed in Table 1. Finally, only the PBL6 (PBS1-like 6, At2G28590) was found to interact with MBD3 (Figure 6, Figure S4). PBL6 was specifically expressed in pollen, which was confirmed with the IP-MS of GFP-tagged PBL6 transgenic plants (Table 2). 

## 3. Discussion

The MBD protein family in plants has been known to act as both “readers” and “erasers” of DNA methylation [30], but the detailed functions of its members are poorly understood. Only a few of them have been reported, and most remain to be characterized. A previous study failed to amplify the RT-PCR products of *AtMBD3* due to its low expression level [22]. In this study, we successfully obtained *mbd3*-mutant plants and found that, despite a low expression level of *AtMBD3,* it played important roles in regulating the plant’s reproductive development. Similarly, *mbd3* knockout in mice proved to be embryonically lethal [23]. Furthermore, we verified the binding capability of methylated and unmethylated DNA sequences of MBD3 protein both in vitro and in vivo.

### 3.1. MBD3 Have the Ability to Bind Sequences More than Methylated DNA

Zemach and Grafi first demonstrated that AtMBD5, AtMBD6, and AtMBD7 can bind the methylated sequences of CG dinucleotides in vitro [14]. Despite its high homology, AtMBD5 can bind both symmetrically methylated CG and asymmetrically methylated CHH sequences, whereas AtMBD6 can only bind methylated CG complexes [31]. Mammalian MBD3 was found not to be able to bind mCG [32]. Our results of AtMBD3, which has a high level of amino acid homology with mammalian MBDs, showed a capability to bind both symmetrically methylated and unmethylated DNA sequences. This result was firstly confirmed both in vitro and in vivo. The capability is more prominent in symmetrically methylated sequences. The ability of MBD5 to bind to mCG may be due to the interaction between conserved 5mCpG residues and residue pairs of R-D and R-E. However, in AtMBD3, residue pairs were changed into R-V and K-E pairs [33]. These substitutions may allow AtMBD3 to not only bind symmetrically methylated DNA sequences, but also guide AtMBD3 to target-specific DNA sequences.

### 3.2. MBD3 Participates in Embryo Development with Other Genes

DNA methylation affecting plant development has been found in many species. The mutation of *OsMET1b* leads to abnormal seed phenotypes in rice, which is associated with either viviparous germination or early embryonic lethality [3,34]. In this study, *MBD3* was also found to be highly expressed in seeds. At the same time, some transcriptional factor genes involved in embryo development were expressed after transcriptome analysis, including *WOX* and *PID* (Figure 5). *WOXs* have been found to make cell-fate decisions during early embryogenesis. *WOX1*, *WOX2*, and *WOX3* were highly expressed in *mbd3* mutants, whereas *WOX8* was not. Both *WOX2* and *WOX8* are initially co-expressed in egg cells and zygotes, and then specifically expressed in apical and basal cell lineages, respectively, after zygotic division [35]. *PID* regulates *PIN* [36], which is responsible for establishing auxin gradients in early embryogenesis [37]. Except for *WOXs* and *PID*, the other expressed genes also showed clear differences between Col-0 and *mbd3* mutants, such as *CUC1, AIB4*, *AGL67*, and *ZOU*. Although *AtMBD3* and these genes are significantly regulated by *MBD3* in *mbd3*-mutant plants, but their relationship has not been confirmed in this paper, it is clear that they coordinately participate in embryo development in some specific manner. 

### 3.3. MBD3 Regulates Pollen and Embryo Development with Other Genes

DNA methylation is also important for the fertility of male plants. Many differentially methylated regions have been identified during tomato fruit development [38]. In *Capsella rubella, NRPD1* knockout leads to the interruption of pollen development at the microspore stage [39]. Pollen formation in *A. thaliana* is associated with the reprogramming of CHH methylation in pollen vegetative cells and a locus-specific restoration in sperm [40]. Our investigation found that *AtMBD3* was expressed in pollen, and it obstructed the development of male germ cell division in the pollen of two *mbd3* mutants. The selected genes in this paper were confirmed to be involved in germ cell development, such as *DUO1, DAZ1,* and *DAZ2,* and also found to be downregulated in the pollen of *mbd3* mutants. Moreover, *mbd3*, *duo1*, and *daz1*/*daz2* double mutants all have problems undergoing fertilization. Therefore, we suspect that *MBD3* and *DUO1* may coordinately play roles in the same pathway. Although *DUO1* and *DAZ2* have no DNA methylation site in genes or promoter regions [41], MBD3 may have the ability to bind them.

PBL6, a specific expressed gene in pollen, was found to directly interact with MBD3 to form a complex. Both PBL6 and MBD3 were also found to interact with MBD5 and MBD6 (Table 2). It has been reported that MBD5 and MBD6 are important in pollen vegetative cell development [42], which indicates that the interaction of MBD3 and PBL6 is important for pollen development, and may interact with MBD5 and MBD6. We hypothesize that through the methylation DNA sequences’ binding ability, MBD3 and PBL6 complex may be recruited into MBD5 and MBD6 binding sites and then impair pollen development. Our study provides direct clues for further research on AtMBD3 and a theoretical basis for a better understanding of AtMBDs family.

## 4. Materials and Methods

### 4.1. Plant Materials and Growth Conditions

*Arabidopsis thaliana* of Columbia-0 (Col-0) ecotype was used as the wild type reference, and all mutant seeds were Col-0 in this study. Seeds were sterilized with 75% ethanol and 5% sodium hypochlorite and then washed three times with 100% ddH_2_O. Sterilized seeds were sown on a 1/2 Murashige and Skoog (1/2 MS) solid medium containing 1% sucrose and 0.7% plant agar powder (*w*/*v*). After being wrapped with aluminum foil at 4 °C for 2 d, the plates were grown at 20–22 °C under cycles of 16 h of light and 8 h of dark in growth chambers. The 2-week-old seedlings were harvested for further experiments or transferred into soil. 

### 4.2. Phylogenetic Analysis of MBD Genes

The search for an HMM profile of the MBD domain (PS50982) in 40 representative species, which are in 11 phyla, including *Rhodophyta, Glaucophyta, Chlorophyta, Streptophyta, Phragmoplastophyta, Marchantiophyta, Bryophyta, Lycopodiophyta, Polypodiophyta, Gymnospermae,* and *Angiospermae* (Table S5), was performed by using the InterProScan software. Phylogenetic trees were constructed in IQTREE software by using maximum likelihood and WAG+R5 with 1000 replicates.

### 4.3. Generation of the CRISPR Allele of mbd3 Mutants 

Given the absence of available T-DNA alleles of *mbd3*, the CRISPR/Cas9 system was used to generate the *mbd3* (AT4G00416) mutant, named as *mbd3-1* and *mbd3-2*. The guide for a 20 bp targeting sequence of sgRNAs was selected from the CRISPR-PLANT platform (https://www.genome.arizona.edu/crispr/CRISPRsearch.html) (accessed on 11 March 2018), and cloned into a YAO promoter-driven CRISPR/Cas9 system in *A. thaliana* [43]. The CRISPR/Cas9 construction was transformed into the *Agrobacterium tumefaciens* strain GV3101, and the floral dip method was used to transform *A. thaliana* Col-0 plants [44]. The T1 transformants were selected on hygromycin plates. After sequencing the AT4G00416 gene from these T1 plants, three of them revealed that they had homozygous deletion, which occurred at the tail of the MBD domain.

### 4.4. ChIP-seq

ChIP-seq experiments were performed by using 2 g of 11-day-old seedlings with biological duplicates on Col-0 and *pJIM(Bar)-GFP-linker::MBD3*/*Col-0* transgenic lines. The samples were fixed in 1% formaldehyde. Then, chromatin was extracted from fixed tissue and fragmented using a Bioruptor^®^ Pico (Diagenode, Belgium) of 200–500 bp fragments. The sheared chromatin was immunoprecipitated overnight by using the following antibodies: anti-GFP (ab290, abcam, Cambridge, dilution 1:100) and antibody IgG (ab6730). Protein A Sepharose beads CL-4B (GE Healthcare, California) were used to capture immunocomplexes. Protein-A beads were washed before use. Chromatin was eluted and de-crosslinked at 65 °C overnight. DNA from immunoprecipitated chromatin was treated with RNase and proteinase K, and then purified by phenol–chloroform extraction and ethanol precipitation. For ChIP-seq, two independent biological replicates of immunoprecipitations were treated to prepare the next-generation sequencing libraries. The Ovation^®^ Ultralow Library Systems (NuGEN, Shanghai) was used for end repair, A-tailing, and the ligation of Illumina-compatible adapters. The data analysis was performed as previously described [45]. The clean reads were mapped to TAIR10 with Bowtie2 and default parameters after using fastqc, multiqc, and trimmomatic to obtain clean reads. Bigwig files were generated by bamCoverage from deeptools, and RPKM was normalized to remove PCR duplication by PICARD. Lastly, the data quality was checked by an integrative genomics viewer (IGV).

### 4.5. Electrophoretic Mobility Shift Assay (EMSA)

MBD3 protein was cloned behind GST (glutathione S-transferase tag). MBD3 was amplified and cloned into bacterial expression vector pGEX-4T-1. The control protein in Figure S2 was cloned behind MBP tag. The construction for expression was transformed into *Escherichia coli* strain BL21 and purified with glutathione Sepharose 4B. A 98 °C water bath was performed on single-stranded nucleotide primers labeled with FAM at the 3′ end, which were used for EMSA, for 10 min and then cooled to room temperature away from light to form labeled double-stranded nucleotides primers. The labeled double-stranded nucleotides primers (160 nM) were incubated with purified AtMBD3 protein or with cold competitors (32 μM) in a total volume of 20 μL reactive system, including 4 μL binding buffer (100 mM Tris-HCl (pH 7.6), 50 mM MgCl_2_, 1% NP40); 5 mM DTT was added before each reaction. Binding reactions were carried out away from light for 20 min, in room temperature, and 1 μL of loading buffer (Beyotime, GS006, Shanghai) was added to each reaction. The protein–substrate complexes were resolved on 6% nondenaturing polyacrylamide gels at 80 V for at least 90 min on ice in a pre-cold 0.5× TBE buffer (44.6 mM Tris, 44.5 mM boric acid, and 1 mM disodium EDTA). After electrophoresis, the gels were detected by using a Typhoon FLA 9500.

### 4.6. Affinity Purification and Mass Spectrometry

The GST-MBD3 fusion protein was used for the binding assays. A total of 2.5 g WT seedlings was ground into fine powder, suspended in 10 mL lysis buffer (50 mM Tris-HCl (pH = 8.0), 230 mM NaCl, 5 mM MgCl_2_, 10% glycerin, 0.2% NP-40, 0.5 mM dithiothreitol (DTT), 1 mM phennylmethysulfonyl fluoride (PMSF) and proteinase inhibitor cocktail tablets (Roche, 14696200)) at 4 °C for 30 min with rotation. Then, 150 μL GST-MBD3 fusion protein was added. After incubation at 4 °C for 3 h, 13,500 g centrifugation for 15 min at 4 °C was performed. The supernatant was incubated with the GST fusion protein beads at 4 °C for 3 h. The bound proteins were boiled in SDS loading buffer after elution with lysis buffer three times, and resolved on 15% denaturing poly-acrylamide gels.

Mass spectrometric identification of the affinity-purified proteins was performed as described previously (Table 2) [46]. Protein bands on the SDS-PAGE were de-stained and in-gel digested with sequencing grade trypsin (0.5 ng/μL). Peptides were extracted by HPLC and sprayed into an LTQ Orbitrap Elite System mass spectrometer (Thermo Scientific, Massachusetts). Database search was performed on an in-house Mascot server (Matrix Science Ltd., London, UK) against the IPI (International Protein Index) *A. thaliana* protein database.

### 4.7. Microscopy Analysis

The morphologies of anther and pollen were observed using cryogenic scanning electron microscopy (cryo-SEM). The dehiscent anthers and pollens were placed on glass slides with double-sided tape, and transferred to the cold stage of chamber under vacuum for sublimation (90 °C, 5 min) after quickly freezing in liquid nitrogen and coated with platinum sputter (10 mA, 30 s) with three repetitions. Finally, the samples were transferred to another cold stage in the scanning electron microscope and imaged. The morphology of anther and pollen was observed under a spinning disc confocal microscope (Zeiss Cell Observer SD; Zeiss, Oberkochen, Germany).

### 4.8. Alexander Dye and 4′,6-Diamidino-2-Phenylindole Staining

An appropriate amount of Alexander dye or 4′,6-diamidino-2-phenylindole (DAPI) was dropped onto glass slides, and the dehiscent anthers were placed on glass slides with dissecting needles. The pollen were gently and quickly suspended in Alexander dye or DAPI, and then the glass slides were covered. After staining, the pollen were observed under the fluorescence microscope (Olympus BX53, Miyazaki). DAPI staining was carried out in the dark.

### 4.9. In Vitro Pollen Germination Analysis

The pollen of newly blooming flowers was collected and placed on the surface of a solid pollen germination medium (SPGM) (18% sucrose, 0.01% boric acid, 2 mM CaCl_2_, 1 mM Ca(NO_3_)_2_, 1 mM KCl, 1 mM MgSO_4_, adjusted to pH = 7, 1.5% agar). The medium was placed in a humid box at 22 °C for at least 6 h of incubation, and then the pollen germination was observed. The ImageJ software was used to measure and calculate the length of the pollens’ tubes and their germination.

### 4.10. RNA Isolation and Quantitative Real-Time RT-PCR (qPCR)

Total RNA was extracted by using Quick RNA isolation kit (Huayueyang, Beijing) and treated with in-column DNase. About 2 μg mRNA was used to amplify the first-strand cDNA by using the superScriptIII First-Strand Synthesis SuperMix Kit. The RT-qPCR was performed using SYBR Green Mastermix (Bio-Rad). An amount of 1 μL cDNA reaction mixture was used as template after dilution in a 20 μL reaction system. The reaction conditions were as follows: firstly, 95 °C for 10 min; secondly, 95 °C for 15 s and 60 °C for 1 min, this step is repeated 40 times; and thirdly, 95 °C for 15 s and 60 °C for 1 min, 95 °C for dissociation. The calculation of the expression level was used as 2^−ΔΔCt^. Two biological replicates from WT and *mbd3* mutants were performed. All analyses of variance were performed in SPSS software by using t-test of the ANOVA program.

Mature pollen were collected from 2 to 3 bunches of unbloomed floral buds from each plant in the same tray. Nine DAP seeds were collected from about 15~20 mature siliques. The fruit pod was removed by using a dissecting needle, after placing siliques on a clean glass slide. The seeds were collected by using tweezers and put in liquid nitrogen for quick freezing. 

### 4.11. Yeast Two-Hybrid Screening

Both pGADT7 vector containing the GLA4 AD and pGBKT7 vector containing the GLA4 BD for the yeast (*Saccharomyces cerevisiae*) were used for two-hybrid assay. BD-MBD3 was constructed by cloning the full-length sequence of MBD3 into the pGBKT7 vector at the NdeI and EcoR1 sites to be fused in-frame with the sequence encoding the GAL4 DNA-binding domain (BD). The *A. thaliana* cDNA library cloned into the prey vector pGADT7-RecAB was constructed by Clontech. The AtMBD3 interaction proteins were screened by using the yeast two-hybrid system, according to the manufacturer’s instructions (Clontech, Matchmaker GAL4 Two-Hybrid System 3 and Libraries User Manual, PT3247-1). The bait plasmid with BD-MBD3 and the prey library DNA were co-transformed into the yeast strain AH109. The resulting progenies were first selected on SD/-Leu/-Trp/-His/-Ade plates, then, β-galactosidase were added for the activity detection to eliminate false positives. Plasmids harboring positive-prey cDNAs were isolated for RT-PCR and sequenced to verify the proteins that interact with AtMBD3.

## Figures and Tables

**Table 1:** Positive proteins selected to screen the interaction with MBD3.

Frequency	ID	Name	Description
13	AT4G25110	ATMC2	Encodes a type I metacaspase.
11	AT2G21620	RD2	Encodes a gene that is induced in response to desiccation
4	AT5G20740		Plant invertase/pectin methylesterase inhibitor superfamily protein
4	AT3G51780	BAG4	Plant homologs of mammalian regulators of apoptosis
3	AT2G28590	PBL6	Protein kinase superfamily protein
2	AT3G18810	PERK6	Encodes a member of the PERK family
2	AT4G39960	DJA5	Molecular chaperone Hsp40/DnaJ family protein
2	AT1G75690	LQY1	DnaJ/Hsp40 cysteine-rich domain superfamily protein
2	AT1G02410	COX11	Encodes a member of the cytochrome c oxidase 11 protein family
2	AT4G17510	UCH3	Ubiquitin C-terminal hydrolase 3
2	AT5G20890	CCT2	TCP-1/cpn60 chaperonin family protein
2	AT4G11260	EDM1	Functions in plant-disease-resistance signaling

‘Frequency’ indicates the number of positive reactions that the protein was identified through RT-PCR.

**Table 2:** Identification of co-purified proteins of GFP-tagged PBL6.

ID	Gene	Description	Unique Peptides
AT2G28590.1	PBL6	Protein kinase superfamily protein	40
AT4G00416.1	MBD3	Methyl-CPG-binding domain 3	7
AT5G59380.1	MBD6	Methyl-CPG-binding domain 6	5
AT3G46580.1	MBD5	Methyl-CPG-binding domain 5	2
AT5G20890.1	CCT2	TCP-1/cpn60 chaperonin family protein	1

## Data Availability

RNA-seq and the ChIP-seq data of *mbd3* have been submitted to the NCBI Sequencing Read Archive under accession no. PRJNA936625. The DNA methylome of Col-0 was previously deposited in SRR005412 [47] and PRJNA686693 [41].

## Supplementary Materials

The following supporting information can be downloaded at: https://www.mdpi.com/article/10.3390/plants12142654/s1, Figure S1: Comparison analysis of MBDs in plantae.; Figure S2: The binding potential of SUVH1 as a positive control.; Figure S3: Deletion of mbd3 has no influence on pollen viability.; Figure S4: Self-activation of MBD3.; Table S1: Primers and sgRNAs used in this study.; Table S2: The oligonucleotide sequences used in this study.; Table S3: ChIP-seq results of MBD3.; Table S4: Genes involved in embryo development. Related to Figure 5; Table S5: Selected genes from RNA-seq results of *mbd3* embryo development. Related to Figure 5; Table S6: Y2H screening results of BD-MBD3.; Table S7: Identification of co-purified proteins of MBD3 by mass spectrometry. GST-MBD3 protein was used for IP/MS.; Table S8: The species involved in phylogenic tree.
