# Supplementary material for: MBD3 Regulates Male Germ Cell Division and Sperm Fertility in Arabidopsis thaliana
Source: Plants (Basel). 2023 Jul 15;12(14):2654. doi: 10.3390/plants12142654 (PMC10384339; doi:10.3390/plants12142654)
Supplement: Supplementary file 1 [file plants-12-02654-s001.zip › plants-2453562-supplementary/SP data/Supplemental Figure legends.pdf]

## Supplementary Figure Legends:

### Figure S1. Comparison analysis of MBDs in plantae.

**(A-B)**The Phylogenetic tree derived from multiple alignments by using IQ-TREE software. Conserved Methyl-CpG-binding domain (MBD) motifs of *Arabidopsis* MBD proteins aligned with the MBD motifs of plantae. MBD proteins in *Arabidopsis* were marked as red rectangle. **(C)** 10 MBD proteins expression pattern from *Arabidopsis* eFP Browser.

### Figure S2. The binding potential of SUVH1 as a positive control.

The binding potential of fusion protein MBP-SUVH1 was used as a positive control to compare with different mCG, mCHG, mCHH and non-methylation sequences. The results were shown the ability of the 3′-Fam-labeled probe to act with AtMBD3 protein. Bound represented the binding band, and free represented free probe.

### Figure S3. Deletion of mbd3 has no influence on pollen viability.

**(A)** Schematic representation of *MBD3* gene structure and the mutation sites. MBD3 only has one conserved MBD domain (marked as blue rectangle), the sgRNA targeting sequences is located in the end of this domain highlighted in yellow. The *mbd3-1* mutation causes a ‘G’ insertion (highlighted in red). The *mbd3-2* mutation causes an eight nucleotide deletion (highlighted in red). Each of these mutations causes a shift in open reading frame. **(B)** Anther morphology of Col-0, *mbd3-1, mbd3-2,* Com #1 and Com #2 plants was observed by using cryo-SEM. Bars = 100 μm. **(C)** Pollen activity of Col-0 and *mbd3-2* plants was detected by Alexander dye staining. The percentages of normal and phenotypically abnormal pollens were recorded both in Col-0 and *mbd3-1* plants. At least 1000 pollen grains were examined in each genotype. Bars = 100 μm. ‘ns’ represent no significant difference (T-test). **(D)** Pollen germination level of Col-0 and *mbd3-2* plants *in vitro*. For each genotype, three times of pollen germination were examined. Bars = 100 μm. ‘ns’ represent no significant difference (T-test).

### Figure S4. Self-activation of MBD3.

The Y2H result showed that MBD3 had no self-activation.

**Table S1. Primers and sgRNAs used in this study.**

**Table S2. The oligonucleotide sequences used in this study.**

Different mCG, mCHG, mCHH and non-methylation sequences The positions of methylated cytosine sites are marked by as ‘Red’.

**Table S3. ChIP-seq results of MBD3.**

The list of ChIP-seq.

**Table S4. Genes involved in embryo development. Related to Figure 5.**

The list including 950 upregulated genes and 1128 downregulated genes changed in wild type Col-0 and *mbd3-2* mutants in tissue embryo, which is important in embryo development.

**Table S5. Selected genes from RNA-seq results of *mbd3* embryo development. Related to Figure 5.**

**Table S6. Identification of co-purified proteins of MBD3 by mass spectrometry. GST-MBD3 protein was used for IP/MS.**

**Table S7. The Species involved in phylogenic tree.**
