# Supplementary material for: MBD3 Regulates Male Germ Cell Division and Sperm Fertility in Arabidopsis thaliana
Source: Plants (Basel). 2023 Jul 15;12(14):2654. doi: 10.3390/plants12142654 (PMC10384339; doi:10.3390/plants12142654)
Supplement: Supplementary file 1 [file plants-12-02654-s001.zip › plants-2453562-supplementary/SP data/Table S1.pdf]

Table S1. Primers and sgRNAs used in this study

Primer name	Sequence (5'-3')	Purpose
MBD3-sg-F	TGAAACCAACGCATAGTAA	CRISPR
MBD3-sg-R	TGTCTTCAATAAACGTTGC	
Cas9-F1	GCTGTTTCTGCTCATTATCCTC	
Cas9-R1	ATTTCCAGTTTTACAAAGTGCG	
U6-26p-F	TGTCCCAGGATTAGAATGATTAGGC	
U6-29p-R	AGCCCTCTTCTTTCGATCCATCAAC	
U6-29p-F	TTAATCCAAACTACTGCAGCCTGAC	
ACTIN2-F	AACTCTCCCGCTATGTATGTCG	Real-time PCR in expression Pattern
ACTIN2-R	AACCCTCGTAGATTGGCACA	
TUBULIN8-F	ATAACCGTTTCAAATTCTCTCTCTC	
TUBULIN8-R	TGCAAATCGTTCTCTCCTTG	
MBD1-F	TCAGAAGTAGAGTACAAGAGGAC	
MBD1-R	TCCTTTCTCAGAATTAAGCTCC	
MBD2-F	GCAGAAGTATGAAGAGATTAGGG	
MBD2-R	TTATCAATAGCCCAAAGACGAG	
MBD3-F	CTCATACGCTGCACAATGTTG	
MBD3-R	ACCATCTTCCTCAGTGTTCCT	
MBD4-F	TTAACTGTCAGAAGAAACAGGG	
MBD4-R	CGGTAGGAGTAAAGTAGTAGGT	
MBD5-F	TGGCACTAAAGCTGGTACTG	
MBD5-R	ATGTGAGTCTCCGTTTTCCG	
MBD6-F	TAGACAGTAGTGCGAAACGACGAC	
MBD6-R	GCGACTTCGATTCCGTCTGAT	
MBD7-F	TCATATATTGAGCCAGGAACAGG	
MBD7-R	CCATAGAAACCGAGTCAAGTGTA	
MBD8-F	GTTCTGATTCAGGGAACCTC	
MBD8-R	GATTCCTGAACTACCAACTCTAC	
MBD9-F	GACATCAAGATGGCCAAACAATG	
MBD9-R	GACTGTCTTATGTCAATTCGTCC	
MBD10-F	AGAAAGACGGAGATTGTGTTTG	
MBD10-R	CAAACTCAGAGATGACAGGATT	
MBD11-F	AGAGTAAGGAGACTGACAAAGA	
MBD11-R	CCATTTTCTTCATGGTTTCTGG	
MBD12-F	GACGTCTATTATTTTCCTCCATCAG	
MBD12-R	GAGAATTGAGAACGATTTACCCC	
MBD13-F	GAAGAATGGGAGAAAGGATAAGG	
MBD13-R	ATATTGCCAGTCTCAACATACC	
DUO1-F	CTTCTCATCGACTCAAGGGC	Real-time PCR in pollen
DUO1-R	ACCTCTTCCTCAACCAAACC	

DAZ1-F	TCGTCTTCTCCAAGGCCTAAAC	
DAZ1-R	TTAGAAGGAGGGTTGATGCCAC	
DAZ2-F	CCGTTGCTTCATCCTCTAGTTC	
DAZ2-R	CCAAGACCAAAACTTTCTGCC	
PRK3-F	GTCTCTGAATACATGCCTAAAAGC	
PRK3-R	TGCCACTCCTTGTATGATCTTC	
PRK6-F	TCCGCCACCCTAACATTTTG	
PRK6-R	TGTTGCCCAAGTTAGCTCG	
MYB97-F	ATCATACAACTCCACTCTCAGC	
MYB97-R	CTCGTTATCTGTTCTGCCTGG	
MYB120-F	GTAACAAATGGGCTCGCATG	
MYB120-R	GGATGGAGTTGATGGTTAGGG	
AIB4-F	CGACTTCGTTTCATCATGAGGTG	Real-time PCR in embryo
AIB4-R	TATAACCCGGATCCAGACCCATA	
AGL67-F	ACATTTGTTGAGAAAGTCCTGCG	
AGL67-R	TTTGAATGATGGGCCGAATTAGC	
EGL3-F	CGATTCAAGCAGCAGAAGTCAAA	
EGL3-R	CTAAGTAGTACCACTCGGTGTCG	
WOX1-F	GTTTCCTCCAACATGTCCAACAG	
WOX1-R	CGTTGGAGCAATCTTCATGTTGT	
WOX2-F	CATAAGAACAACAGCAGCCACAA	
WOX2-R	GGAAAAAGAGGGAGTGTTTTCCG	
WOX3-F	GTGCAGATACAACAGATCACAGC	
WOX3-R	GGCTAGTTTCTTCCTCAGCTTCT	
ZOU-F	GCCTCAACAAACACAACTCCTAC	
ZOU-R	GGCACTGGAAGAAGATACCTGAT	
CUC1-F	AAGCCACTGGTAAAGACAGAGAG	
CUC1-R	ATGACCCAACAACTCTTCTCTCC	
PID-F	CCATCCATTTTTGCCGACTCTTT	
PID-R	ACGGAGAGAGTGTAAATCACCAC	
KAN2-F	GAGAGATAGCGAGGAATTGACGA	
KAN2-R	TCGATTAACTTCCCATGCAAACG	
WOX8-F	TGGTTTCAAAACCGGAAATCTCG	
WOX8-R	CCAAAACAATGATCAGCGTCAGT	
MBD3-F	GTTTATTGAAGACAAGGAAGA	PCR
MBD3-R	TAGGTAGCAGTTTGTTCAGAG	
pGKAD/pGKBD-T7-F	TAATACGACTCACTATAGGGCG	
pGKAD-T7-R	AGATGGTGCACGATGCACA	
GFP-F	TGCACTACTGGAAAACTACC	
GFP-R	TCCTTGAAGAAGATGGTCCT	
