# Supplementary material for: MBD3 Regulates Male Germ Cell Division and Sperm Fertility in Arabidopsis thaliana
Source: Plants (Basel). 2023 Jul 15;12(14):2654. doi: 10.3390/plants12142654 (PMC10384339; doi:10.3390/plants12142654)
Supplement: Supplementary file 1 [file plants-12-02654-s001.zip › plants-2453562-supplementary/SP data/Table S2.pdf]

Table S2. The oligonucleotide sequences used in this study

Methylation type	Oligonucleotide sequences
mCG-F	TCAATGACGCTACTGGTCGCTGCTTCTGCAACGGATACT
mCG-R	AGTATCCGTTGCAGAAGCAGCGACCAGTAGCGTCATTGA
mCHG-F	TCAATGACGCTACTGGTCGCTGCTTCTGCAACGGATACT
mCHG-R	AGTATCCGTTGCAGAAGCAGCGACCAGTAGCGTCATTGA
mCHH-F	TCAATGACGCTACTGGTCGCTGCTTCTGCAACGGATACT
mCHH-R	AGTATCCGTTGCAGAAGCAGCGACCAGTAGCGTCATTGA
unmethylated-F	TCAATGACGCTACTGGTCGCTGCTTCTGCAACGGATACT
unmethylated-R	AGTATCCGTTGCAGAAGCAGCGACCAGTAGCGTCATTGA

Red marks as methylation sites
