# Supplementary material for: MBD3 Regulates Male Germ Cell Division and Sperm Fertility in Arabidopsis thaliana
Source: Plants (Basel). 2023 Jul 15;12(14):2654. doi: 10.3390/plants12142654 (PMC10384339; doi:10.3390/plants12142654)
Supplement: Supplementary file 1 [file plants-12-02654-s001.zip › plants-2453562-supplementary/SP data/Table S5.pdf]

Table S5. Selected genes from RNA-seq results of *mbd3* embryo development

Molecular encoded	Gene model name	Primary gene symbol	Change	Function	Involved stage
NAC domain txn factor	AT3G15170	CUC1	Down	Cotyledon boundary formation; act synergistically with STM to control SAM formation	Stage1-morphogenesis
MADS box	AT1G77950	AGL67	Up	Desiccation tolerance	Stage1-morphogenesis
NF-YB	AT1G21970	LEC1	Down	Embryonic fate; regulates storage protein and lipid accumulation; act as a molecular signal between endosperm and embryo; embryo maturation; desiccation tolerance	Stage1-morphogenesis
AP2	AT2G40220	ABI4	Up	Seed dormancy; ABA signalling pathway	Stage2-maturation
bHLH txn factor	AT1G63650	EGL3	Up	Redundant with GL3 and TT8 and interacts with TTG1	Stage2-maturation
bHLHtxn factor	AT1G49770	ZOU	Down	Seed dormancy	Stage2-maturation
GARP/KAN	AT1G32240	KAN2	Up	Abaxial patterning	Stage1-morphogenesis
Homebox txn factor	AT3G18010	WOX1	Up	Together with WOX2 regulate apical patterning and establishment of SAM	Stage1-morphogenesis

Homebox txn factor	AT5G59340	WOX2	Up	Restricted to apical daughter following division; development of SAM	Stage1-morphogenesis
Homebox txn factor	AT2G28610	WOX3	Up	Together with WOX2 regulate apical patterning and establishment of SAM	Stage1-morphogenesis
Homebox txn factor	AT5G45980	WOX8	Down	Restricted to basal daughter following division; zygotic polarity	Stage1-morphogenesis
serine/threonine kinase	AT2G34650	PID	Up	Negative regulator of auxin signaling	Stage1-morphogenesis
