# Supplementary material for: MBD3 Regulates Male Germ Cell Division and Sperm Fertility in Arabidopsis thaliana
Source: Plants (Basel). 2023 Jul 15;12(14):2654. doi: 10.3390/plants12142654 (PMC10384339; doi:10.3390/plants12142654)
Supplement: Supplementary file 1 [file plants-12-02654-s001.zip › plants-2453562-supplementary/SP data/Table S7.pdf]

Table S7. Identification of co-purified proteins of GST-MBD3 by using mass spectrometry. Col-0 plants grown under normal growth conditions were used for IP/MS.

ID	Gene	Description	Unique peptides
AT4G00416.1	MBD3	methyl-CPG-binding domain 3	7
AT4G33470.1	MBD2	histone deacetylase 14	5
AT5G59380.1	MBD6	methyl-CPG-binding domain 6	1
AT5G20890	CCT2	TCP-1/cpn60 chaperonin family protein	1
