# Supplementary material for: MBD3 Regulates Male Germ Cell Division and Sperm Fertility in Arabidopsis thaliana
Source: Plants (Basel). 2023 Jul 15;12(14):2654. doi: 10.3390/plants12142654 (PMC10384339; doi:10.3390/plants12142654)
Supplement: Supplementary file 1 [file plants-12-02654-s001.zip › plants-2453562-supplementary/SP data/Table S8.pdf]

Table S7. The species involved in phylogenic tree

Phylum	Class	Order	Family	Species	Data type	Link
Rhodophyta	Cyanidiophyceae	Cyanidiales	Cyanidiaceae	*Cyanidioschyzon merolae*	Genome	https://www.ncbi.nlm.nih.gov/genome/79
				Galdieria sulphuraria	Genome	https://www.ncbi.nlm.nih.gov/genome/405
	Florideophyceae	Gigartinales	Gigartinaceae	*Chondrus crispus*	Genome	https://www.ncbi.nlm.nih.gov/genome/12016
	Bangiophyceae	Bangiales	Bangiaceae	*Porphyra umbilicalis*	Genome	https://www.ncbi.nlm.nih.gov/genome/12861
Glaucophyta	Glaucocystophyceae	Glaucocystales	Cyanophoraceae	*Cyanophora paradoxa*	Genome	https://www.ncbi.nlm.nih.gov/genome/303
Chlorophyta	Mamiellophyceae	Mamiellales	Bathycoccaceae	*Ostreococcus lucimarinus*	Genome	https://www.ncbi.nlm.nih.gov/genome/373
				Bathycoccus prasinos	Genome	https://www.ncbi.nlm.nih.gov/genome/12309
			Mamiellaceae	*Micromonas pusilla*	Genome	https://www.ncbi.nlm.nih.gov/genome/501
	Trebouxiophyceae	T.ordo incertae sedis	Coccomyxaceae	*Coccomyxa subellipsoidea*	Genome	https://www.ncbi.nlm.nih.gov/genome/2692
		Chlorellales	Chlorellaceae	*Chlorella variabilis*	Genome	https://www.ncbi.nlm.nih.gov/genome/694
				Auxenochlorella protothecoides	Genome	http://plantregmap.cbi.pku.edu.cn/
	Ulvophyceae	Ulvales	Ulvaceae	*Ulva mutabilis*	Genome	https://www.ncbi.nlm.nih.gov/genome/72458
	Chlorophyceae	Chlamydomonadales	Chlamydomonadaceae	*Chlamydomonas reinhardtii*	Genome	https://phytozome.jgi.doe.gov/
			Dunaliellaceae	*Dunaliella salina*	Genome	http://plantregmap.cbi.pku.edu.cn/
			Volvovaceae	*Volvox carteri*	Genome	http://plantregmap.cbi.pku.edu.cn/
				Gonium pectorale	Genome	https://www.ncbi.nlm.nih.gov/genome/16856

		Sphaeropleales	Selenastraceae	*Monoraphidium neglectum*	Genome	https://www.ncbi.nlm.nih.gov/genome/36372

**Phylum**	**Class**	**Order**	**Family**	**Species**	**Data type**	**Link**
Streptophyta	Mesostigmatophyceae	Mesostigmatales	Mesostigmataceae	*Mesostigma viride*	Genome	https://www.ncbi.nlm.nih.gov/geo/query/acc.cgi?acc=GSE123852
	Klebsormidiophyceae	Klebsormidiales	Klebsormidiaceae	*Klebsormidium nitens*	Genome	https://www.ncbi.nlm.nih.gov/genome?term=Klebsormidium
Phragmoplastophyta	Charophyceae	Charales	Characeae	*Chara braunii*	Genome	https://bioinformatics.psb.ugent.be/orcae/
	Zygnematophyceae	Zygnematales	Mesotaeniaceae	*Mesotaenium endlicherianum*	Genome	https://www.ncbi.nlm.nih.gov/genome/33366
		Spirogloeales	Spirogloeaceae	*Spirogloea muscicola*	Genome	https://www.ncbi.nlm.nih.gov/genome/86225
Marchantiophyta	Marchantiopsida	Marchantiales	Marchantiaceae	*Marchantia polymorpha*	Genome	https://www.ncbi.nlm.nih.gov/genome/3220
Bryophyta	Bryopsida	Funariales	Funariaceae	*Physcomitrella patens*	Genome	https://www.ncbi.nlm.nih.gov/genome/383
	Sphagnopsida	Sphagnales	Sphagnaceae	*Sphagnum fallax*	Genome	https://phytozome.jgi.doe.gov/
Lycopodiophyta	Lycopodiopsida	Selaginellales	Selaginellaceae	*Selaginella moellendorffii*	Genome	https://www.ncbi.nlm.nih.gov/genome/411
Polypodiophyta	Polypodiopsida	Salviniales	Salviniaceae	*Azolla filiculoides*	Genome	https://www.fernbase.org/
				Salvinia cucullata	Genome	https://www.fernbase.org/
Gymnospermae	Pinopsida	Pinales	Pinaceae	*Picea abies*	Genome	https://www.ncbi.nlm.nih.gov/genome/11155
	Ginkgoopsida	Ginkgoales	Ginkgoaceae	*Ginkgo biloba*	Genome	http://gigadb.org/dataset/100209
	Gnetopsida	Gnetales	Gnetaceae	*Gnetum montanum*	Genome	https://datadryad.org/resource/doi:10.5061/dryad.0vm37.2
Angiospermae	basal Angiosperms	Amborellales	Amborellaceae	*Amborella trichopoda*	Genome	http://www.angiosperms.org/
	Monocotyledoneae	Poales	Poaceae	*Oryza sativa*	Genome	http://www.angiosperms.org/

			Zea mays	Genome	http://www.angiosperms.org/
	Asparagales	Asparagaceae	*Asparagus officinalis*	Genome	http://www.angiosperms.org/
Eudicotyledoneae	Ranunculales	Ranunculaceae	*Aquilegia coerulea*	Genome	http://www.angiosperms.org/
	Brassicales	Brassicaceae	*Arabidopsis thaliana*	Genome	http://www.angiosperms.org/
	Malpighiales	Salicaceae	*Populus deltoides*	Genome	http://www.angiosperms.org/
	Asterales	Asteraceae	*Helianthus annuus*	Genome	http://www.angiosperms.org/
	Solanales	Solanaceae	*Solanum lycopersicum*	Genome	http://www.angiosperms.org/
